# Position paper on the existing selection procedure in Germany for the degree program human medicine – interim evaluation after four years. Position paper of the student selection (ASA) in the DACH Association for Medical Education (GMA)

**DOI:** 10.3205/zma001820

**Published:** 2026-02-17

**Authors:** Brigitte Müller-Hilke, Kirsten Gehlhar

**Affiliations:** 1 University Medical Center Rostock, Core Facility for Cell Sorting and Cell Analysis and Research Group Clinical Immunology, Rostock, Germany; 2 Carl von Ossietzky University of Oldenburg, School VI Medicine and Health Sciences, Office of Student Affairs, Oldenburg, Germany

**Keywords:** selection procedure, student selection, SJT, MMI, school grade, HamNat, TMS, fairness

## Abstract

Four years after the entry into force of the new allocation rules, this position paper by the Committee on Student Selection of the GMA presents an initial interim assessment of the intended aspects of aptitude, public welfare considerations, and the social state principle, while also addressing equality-based admission. Academic achievement continues to be used as the primary selection criterion, even though it only moderately predicts success in the first phase of study and tends to favor both socioeconomically advantaged applicants and female high school graduates. HamNat and TMS results increase the predictive validity of the school-leaving grade and, in particular, improve the chance of admission for male applicants. Consideration of public welfare aspects is only partially achieved in view of the preferential allocation of study places to federal states with good medical care. Immediate measures in response to already apparent consequences should include: 1) a revision of the state treaty in order to i) reconcile individual fairness with a needs-based distribution of study places to state residents in the algorithm for the federal list based on school-leaving grades, and ii) enable the tracking of individual study trajectories to evaluate the effects of selection criteria and, if necessary, make adjustments; 2) examining whether a combination of HamNat and TMS further improves the predictive validity for study success compared to using only one of them; 3) giving the school-leaving grade less weight in the allocation of study places in favor of HamNat and/or TMS, to increase gender equality and favor socioeconomically disadvantaged applicants, thereby enhancing diversity within the student body; 4) ensuring that the criterion of fairness is examined and weighed in all test procedures considered in student selection.

This could promote diversity in medical education and lead to a future medical profession that continues to adequately represent and serve the entire population.

## Abstract (de)

Vier Jahre nach Inkrafttreten der neuen Vergaberegeln zieht das vorliegende Positionspapier des Ausschusses Studierendenauswahl der GMA eine erste Zwischenbilanz zu den intendierten Aspekten der Eignung, Gemeinwohlbelange und Sozialstaatprinzip und thematisiert dabei auch die gleichheitsgerechte Zulassung. Nach wie vor werden schulische Leistungen als primäres Auswahlkriterium herangezogen, obwohl diese nur etwas über den Erfolg im ersten Studienabschnitt vorhersagen und sowohl sozioökonomisch besser Gestellte, als auch weibliche Abiturienten begünstigen. HamNat- und TMS-Ergebnisse erhöhen die prädiktive Validität der Abiturnote und verbessern vor allem bei männlichen Bewerbern die Chance auf einen Studienplatz. Die Berücksichtigung von Gemeinwohlbelangen wird vor dem Hintergrund einer bevorzugten Allokation von Studienplätzen auf Länder mit guter medizinischer Versorgung nur bedingt erfüllt. Unmittelbare Maßnahmen aufgrund bereits jetzt ersichtlicher Konsequenzen sollten 1) eine Überarbeitung des Staatsvertrags beinhalten, um i) beim Algorithmus für die Bundesliste nach Abiturnoten die individuelle Gerechtigkeit mit einer bedarfsgerechten Verteilung von Studienplätzen auf Landeskinder in Einklang zu bringen, und ii) die Nachverfolgung individueller Studienverläufe zu ermöglichen, um die Wirkung der Auswahlkriterien evaluieren und ggf. nachbessern zu können. 2) Eine Kombination aus HamNat und TMS sollte daraufhin untersucht werden, ob sie die Vorhersagekraft für den Studienerfolg gegenüber einem einzelnen Einsatz weiter verbessert. 3) Die Abiturnote sollte bei der Vergabe von Studienplätzen zu Gunsten von HamNat und/oder TMS eine geringere Gewichtung erfahren, um durch mehr Gendergerechtigkeit und Begünstigung sozioökonomisch Benachteiligter die Diversität innerhalb der Studierendenschaft zu erhöhen. 4) Das Kriterium der Fairness sollte bei allen Testverfahren, die bei der Studierendenauswahl berücksichtigt werden, untersucht und abgewogen werden. Dadurch könnte die Vielfalt im Medizinstudium gefördert und eine zukünftige Ärzteschaft hervorgebracht werden, die die gesamte Bevölkerung auch zukünftig angemessen repräsentiert und versorgt.

## Introduction

The study of human medicine has been one of the most sought-after degree programs for decades in German-speaking and Anglo-Saxon countries, with the number of applicants exceeding the number of available study places by approximately a factor of four. In Germany, during the summer semester 2025 and the winter semester 2024/25 combined, a total of 12,030 study places were available for 45,724 applicants (data from SfH, April 4, 2025, via MFT). In light of the constitutional right to free choice of profession, a selection process must therefore be carried out that – according to the decision of the Federal Constitutional Court of December 2017 – must primarily be based on the criterion of aptitude. According to statistics from the German Medical Association, by the end of 2023 around 428,000 practicing physicians were responsible for the health of approximately 83 million citizens in Germany [1]. This responsibility is significant and demands high standards of the medical profession – but it is also costly. In 2018, more than €391 billion were spent on health care [2]. The costs for studying human medicine amount to nearly €200,000 up to the third state examination (6.25 years), meaning that around €400 million are spent annually for the education of about 12,000 physicians [3]. Against this background, the demand to admit only those who demonstrate the appropriate aptitude is understandable.

With its ruling of December 2017, the Federal Constitutional Court required a reform of the allocation procedure for medical study places [4]. These new regulations came into force in the summer semester 2020, abolishing the waiting-time quota and allowing applicants to indicate unrestricted location preferences, which are relevant for the assignment of study locations but not for admission itself. Additionally, an extra aptitude quota (ZEQ) of 10% of study places was introduced, in which the school-leaving grade is not considered, and finally a compensation mechanism for state-specific differences in school-leaving grades was implemented. What has not changed, however, is the sole importance of the higher education entrance qualification (HZB) grade in the “best school grade” quota, which was increased to 30% (previously 20%) of study places. For the remaining study places within the university selection procedure (AdH), additional selection criteria – such as aptitude tests (TMS or HamNat), medical-related vocational training, or federal voluntary service – were made mandatory alongside the school grade. These criteria are used by all faculties, albeit with different weightings; elaborate on-site selection procedures, however, are rarely conducted anymore (primarily due to administrative reasons). At the same time, the Court’s 2017 decision required that, in addition to the right to equality-based admission to higher education, the rules governing the distribution of scarce study places must be fundamentally oriented toward the criterion of aptitude, must take public welfare considerations into account, and must comply with the social state principle [4].

Four years after the new allocation rules took effect, it is still too early to fully assess their impact on faculties, final examinations, or graduates. Nonetheless, this position paper by the Committee on Student Selection provides an initial interim evaluation of the intended aspects of aptitude, public welfare considerations, and the social state principle, while addressing equality-based admission.

## The aspect of aptitude

The Federal Constitutional Court’s ruling of 2017 does not define which type of aptitude should actually be considered: the aptitude to successfully complete the degree program with good grades and within the standard period of study? Or the aptitude to later practice the profession professionally and competently? The latter, in turn, refers to quite different competencies depending on which type of medical professionals are being sought. Should communication or teamwork skills be prioritized? Are scholars being sought? Managers? Physicians working in curative care or those employed outside clinical settings? What defines a good physician? And would it be appropriate to expect the desired competencies of the future profession to already be present among applicants for a study place? Should they not rather be developed during the course of study? And how realistic is it to assume that the characteristics and personalities identified in high-school graduates remain unchanged after completing medical education [5], [6]?

### Focus on academic success

In order to avoid both the criterion problem [7], [8], [9] – that is, the challenge of capturing aptitude for all potential professional fields through a single selection criterion – and the inability to predict professional behavior at the time of application, the current selection procedure focuses mainly on aptitude for successful completion of studies. Indeed, academic performance already serves as an important indicator of a student’s ability to meet the demanding requirements of medical education. Accordingly, school or college grades have been established worldwide as a primary selection criterion for medical programs [10], [11]. However, pre-university academic performance explains only between 20% and 30% of the variance in academic achievement, and even then, only for the first study phase [10], [11], [12], [13].

Nevertheless, the higher education entrance qualification (HZB=school leaving grade) remains an important predictor of study success in Germany, since in traditional medical programs the preclinical phase ends with the first part of the state medical examination – the *Physikum* – which constitutes the main hurdle of the study program. After this, in the clinical phase, students rarely drop out [11], [12]. The relatively low dropout rate in human medicine – around 6% – is also attributed to the restricted access due to the numerus clausus system [13].

However, the HZB grade includes several ambiguities: it provides little information about deviations from the standard period of study, fails to consider average school or state-specific grading differences, and does not distinguish between natural and social science subject combinations in the high-school diploma [11]. Furthermore, the HZB grade carries a gender bias: girls generally achieve better grades, and with only 53% of boys among 18-year-olds, they now occupy about 66% of study places. Even though the reasons for this performance difference are unclear, it prevents an equality-based admission process [14].

### Aptitude tests as an addition

The steadily increasing number of high-school graduates with top grades makes it clear that the higher education entrance qualification alone is no longer sufficient to provide clear differentiation. Therefore, it becomes necessary to develop more suitable or additional selection criteria. Worldwide, so-called aptitude tests – such as the UCAT in the United Kingdom, the MCAT in the United States, the Qudraat in Saudi Arabia, and the TMS in Germany – have become established tools. These tests assess cognitive abilities such as concentration, reading comprehension, spatial reasoning, or numerical problem-solving – usually without requiring specific subject knowledge but focusing instead on fluid intelligence [15], [16].

The TMS, for instance, correlates moderately with school grades on its verbal-mathematical items, but not at all on its figural-spatial components – indicating that it measures additional cognitive abilities [17]. Studies show that simply participating in an aptitude test is associated with higher academic success and lower dropout rates [18], [19], [20].

A British longitudinal study with more than 3,000 graduates even found significant correlations between UCAT and BMAT results and the successful completion of the medical licensing examinations (MRCP) – in the first two knowledge-based parts as well as the third practical part. These tests demonstrated higher predictive validity than school grades, underlining the importance of problem-solving abilities in the medical profession [21].

### Interaction between HZB and test results

A combination of school grades and test performance – particularly when using the HamNat – shows the best predictive power in Germany for passing the *Physikum* after seven semesters and for lower dropout rates [11], [22], [23], [24]. Studies from the UK support this finding: the scientific component of the BMAT predicts early academic success better than the reasoning component [25].

Against this background, further investigation of a combination of TMS (aptitude) and HamNat (knowledge) appears promising and should determine whether this combination improves predictive validity for study success compared to the use of only one test. However, this requires consistent longitudinal tracking of the students through to the third state examination and beyond, which should be incorporated into the state treaty on student selection.

### Non-cognitive and metacognitive factors: Assessment, potential, and limitations

Personality traits – particularly conscientiousness and its facets of dutifulness, self-discipline, and striving for achievement – have been shown to contribute more strongly to academic success than intelligence alone [10], [26], [27], [28]. They foster motivation and structured preparation, which becomes indirectly relevant in tests such as HamNat and BMAT that emphasize scientific knowledge.

Moreover, metacognitive learning strategies (e.g., systematic planning, self-monitoring, adaptive regulation) act as mediators between personality and performance [29], [30], [31]. Research demonstrates that self-efficacy and intrinsic motivation enhance learning satisfaction and exam preparation [32], [33], [34], [35]. Subject-specific interest – e.g., in the natural sciences – is likewise associated with better performance [29], [30], [36], [37], [38].

To assess social and personal competencies such as empathy, integrity, or teamwork, Multiple Mini-Interviews (MMIs) and Situational Judgment Tests (SJTs) have been developed [13], [39], [40], [41], [42], [43]. Studies confirm that MMIs are superior to traditional interviews [44], [45] and that their results correlate with later clinical performance [45], [46], [47]. SJTs likewise show correlations with training evaluations [48] and are considered comparatively gender- and status-neutral [49]. However, these methods also include cognitive components [45], [50], [51] and require high methodological standards in development and administration [41], [49].

There is a need for the development of an instrument that can be implemented nationwide and that allows resource-adequate assessment of social and personal competencies.

### Other selection criteria

Motivation letters, letters of recommendation, and interviews, according to meta-analyses, have only low predictive validity [13] and are therefore rarely used in Germany. In contrast, previous medical-related vocational training is increasingly considered as a non-cognitive selection criterion. Initial multicenter studies from Germany indicate a slightly positive predictive value [17]. Possible advantages include greater resilience or better integration of prior knowledge [52], [53], although the higher age of applicants may also entail disadvantages (e.g., apparent in the abolished waiting-time quota).

Thus, the current selection practice in medical education represents a pragmatic model primarily oriented toward predicting academic success. This focus is understandable, as other relevant dimensions of aptitude – particularly profession-related competencies – are not yet validly or efficiently measurable. Nonetheless, there is a growing evidence-based need to diversify the selection process through:

- combining existing cognitive tests (e.g., TMS, HamNat),
- integrating non-cognitive procedures (e.g., MMI, SJT), and
- considering personality traits and learning strategies.

This would allow student selection to be not only performance-oriented but also profession-related and fairer. However, this requires the implementation of prospective longitudinal studies and structural anchoring of appropriate instruments in the legal framework.

### Public welfare considerations

Public welfare (Gemeinwohl) is enshrined in the German constitutional law as a guiding principle of state action [https://www.gesetze-im-internet.de/gg/BJNR000010949.html]. It encompasses all aspects that benefit the majority of people in a community or state – including, among others, ensuring comprehensive medical care. The current selection procedure for medical studies affects this aspect in problematic ways: The introduced federal-state compensation mechanism in the admission process is intended to protect applicants from states with comparatively lower average high-school grades from being disadvantaged. Two factors are incorporated into the calculation of the nationwide ranking:

- the propotion of applicants from each federal state relative to the total number of applicants, and
- the proportion of 18- to 21-year-olds in the overall population [6].

Assuming an equal distribution of applicant shares, this results in an implicit allocation of study places roughly according to the population size of each state, with city-states receiving a 30% premium.

In reality, however, structural differences exist: the new federal states have a smaller proportion of young people (3.4% vs. 4.4%) and a larger proportion of elderly citizens (33% vs. 27%) [54]. The proportion of applicants among 18- to 21-year-olds is also significantly lower there (1.5% vs. 1.8-3.6%) [46]. At the same time, a higher percentage of high-school graduates in these states achieve top grades [55].

The consequence: federal states that already have fewer applicants, suffer from medical under-provision, and have an above-average share of elderly citizens lose additional study places through the current calculation method [56]. Because physicians’ later practice location correlates strongly with their region of origin [57], this allocation procedure risks further worsening medical care in structurally weak regions – thus impairing the public welfare in a particularly significant way.

Political counter-measures include pre-admission quotas (e.g., the *Landarztquote* for rural doctors, or quotas for the public health service). However, these measures fall outside the faculties’ selection criteria and the Constitutional Court’s framework. Whether they truly serve public welfare remains uncertain.

A possible adjustment of the selection process could include, in addition to the number of 18- to 21-year-olds, the regional medical care situation (e.g., physician density or proportion of residents over 60) in the allocation formula. While political authorities ultimately bear responsibility for ensuring medical care, a more targeted allocation of study places to state residents from underserved regions could help prevent the shortage of doctors from becoming even more acute.

### The principle of the social state

The principle of the social state obliges the government to ensure social balance – particularly between socially privileged and disadvantaged groups. This also applies to the allocation of study places. Against this background, the question arises as to what extent the current selection process truly provides equal opportunities and grants all applicants – regardless of gender, social background, or cultural capital – fair access to medical studies.

In 2017, the legislature stipulated that the allocation of scarce study places must primarily be based on the criterion of aptitude. At the same time, the right to equality-based admission must be preserved. In practice, however, structural distortions occur.

For example, common aptitude tests such as HamNat and TMS systematically favor applicant groups that already have advantageous starting conditions: men, applicants over 21 years of age, native German speakers, or those from academic households perform better in the HamNat [58]. For the TMS, it has been shown that men with lower school grades more often can compensate these grades with good test results, thereby improving their chances of admission – an effect confirmed in a larger cohort [59], [60].

Similar patterns can be observed in international admission tests such as EMS (Switzerland), MedAT (Austria), UKCAT (United Kingdom), and SAT/ACT (United States) [61], [62], [63], [64], [65]. In Germany, the introduction of an aptitude test for psychology in Berlin led to a sharp increase in the proportion of male students (from 25% to 45%) [66]. Since women, on average, achieve better school grades, aptitude tests tend to create a more balanced gender ratio – yet they also introduce new mechanisms of social selection. Therefore, the school-leaving grade should be given less weight in favor of HamNat and/or TMS to promote gender equality and advantage socioeconomically disadvantaged applicants, thereby increasing diversity within the student body. Socioeconomic status has a particularly strong influence: students from low-income or non-academic families apply less frequently for medical studies [67], [68], [69], perform worse in both school-based and test-based selection procedures [41], [48], [65], [70], [71], [72], [73], [74], [75], and are underrepresented among medical students [76], [77], [78]. This results in a relatively homogeneous physician workforce [76] that neither socially nor culturally reflects the population it serves — with potential consequences for the quality of healthcare delivery [79].

To counteract this, applicants from disadvantaged groups should be supported through targeted and low-threshold measures: early information and counseling at schools, free preparatory courses to facilitate access, and academic support during their studies. These steps would help ensure that the intended improvement in equal opportunity – achieved by placing greater weight on TMS or HamNat – can be realized effectively.

Internationally, this is increasingly discussed under the concept of the “social responsibility of medical faculties” [8], [67], [80], [81], [82]: disadvantaged groups should be actively approached, supported during the admission process, and accompanied throughout their studies. The goal in Germany, too, should be to develop an admission system that maintains a balance between academic aptitude and equal opportunity. Faculties already have flexibility within the AdH (university selection quota), for instance through the adaptable weighting of the HZB grade or the inclusion of additional criteria.

In the long term, transparent evaluations are needed to determine which combinations of selection criteria produce what kind of student body – so that timely adjustments can be made if necessary. The criterion of fairness should also be examined and weighed for all test procedures used in student selection.

## Conclusion & recommendations

The new selection procedure for students of human medicine has now been in place for four years, allowing for an initial assessment of the requirements set out in the Constitutional Court’s ruling from December 2017. The following points are clearly positive: the unrestricted number of location preferences and greater individual fairness achieved through the state-wide equalization of high school graduation grades are clearly positive developments. Nevertheless, the current selection procedure should be regarded only as an interim solution that requires continuous and close evaluation.

We identify fundamental problem areas in the current selection process:

1. The increased weighting of the school-leaving grade at the expense of TMS and/or HamNat prevents gender equality and disadvantages socioeconomically less privileged applicants, meaning that true diversity within the student body is not ensured.
2. The criteria currently used in student selection have not been sufficiently examined for fairness.
3. Aptitude continues to be defined primarily in terms of academic performance in the first phase of study because scientific evidence for alternative selection criteria is lacking.
4. Public welfare considerations are jeopardized by the specific algorithm used in the federal-state adjustment of school-leaving grades, as the current model – beyond compensating for grade differences – leads to a redistribution of study places to the detriment of the new federal states.

As a result, the shortage of medical professionals in underserved regions is likely to intensify further in the long term.

We therefore propose that the state treaty governing student selection should be revised in two key respects:

- Longitudinal study trajectories should be traceable, and research findings should be made accessible. Only then can well-founded recommendations for alternative selection criteria be made. Experiences from the stav project show that data protection–compliant implementations are possible. International examples such as HESA (Higher Education Statistics Agency, [https://www.hesa.ac.uk/]) illustrate the potential of centralized solutions, but represent an organizational scale that we do not wish to anticipate at this point.
- The calculation algorithm for the national ranking list of school-leaving grades should be modified so that, instead of the proportion of 18- to 21-year-olds in each federal state, the proportion of individuals over 60 years of age is taken into account. By giving greater consideration to state residents when allocating study places, graduates could be more likely to remain in underserved regions after completing their studies.

## Notes

### Statement

This position paper was agreed upon by the members of the Student Selection Committee at the committee meeting on June 10, 2024, in the form submitted on October 16, 2024. The members of the committee at that time were: PD Dr. Volkart Fischer, Dr. Kirsten Gehlhar, Prof. Wolfgang Hampe, Prof. Brigitte Müller-Hilke, Prof. Holger Repp.

### Adoption

The position paper was adopted by the GMA executive board on November 26, 2025.

### Authors’ ORCIDs

- Brigitte Müller-Hilke: [0000-0002-6575-6824]
- Kirsten Gehlhar: [0009-0003-1456-9046]

## Competing interests

The authors declare that they have no competing interests. 
